# Understanding Bayesian analysis of clinical trials: an overview for clinicians

**DOI:** 10.62675/2965-2774.20250267

**Published:** 2025-05-13

**Authors:** Callum Taylor, Kathryn Puxty, Tara Quasim, Martin Shaw

**Affiliations:** 1 University of Glasgow Glasgow United Kingdom University of Glasgow - Glasgow, United Kingdom.; 2 NHS Greater Glasgow and Clyde Glasgow United Kingdom NHS Greater Glasgow and Clyde - Glasgow, United Kingdom.

**Keywords:** Bayesian analysis, Statistical analysis, Methodology, Research

## Abstract

Bayesian analysis is being used with increasing frequency in critical care research and brings advantages and disadvantages compared to traditional Frequentist techniques. This study overviews this methodology and explains the terminology encountered when appraising this literature. Setting different priors can impact the interpretation of new results, and we describe an approach to understanding this. Finally, the strengths and challenges of adopting a Bayesian analysis compared to Frequentist techniques are explored.

## INTRODUCTION

Bayesian analysis is a theory in the field of statistics that allows us to numerically describe our estimated belief in a hypothesis before testing how strongly we hold this belief and update that belief after we receive new information. It has many advantages and some disadvantages over traditional Frequentist statistics and is increasingly being deployed in the medical literature.^(1)^ Bayesian statistics offer an alternative framework for data interpretation that provides the probability of a hypothesized treatment effect utilizing all available data. As Bayes´s approach becomes more commonplace across the critical care literature,^(2-6)^ readers need to understand this technique. This study introduces the components involved in Bayes, provides the reader with the knowledge required to read and critique a Bayesian clinical trial, and compares the perceived advantages and disadvantages of Bayesian techniques to Frequentist approaches.

### Concepts of Bayesian statistics

#### Parameters

**Figure 1:**
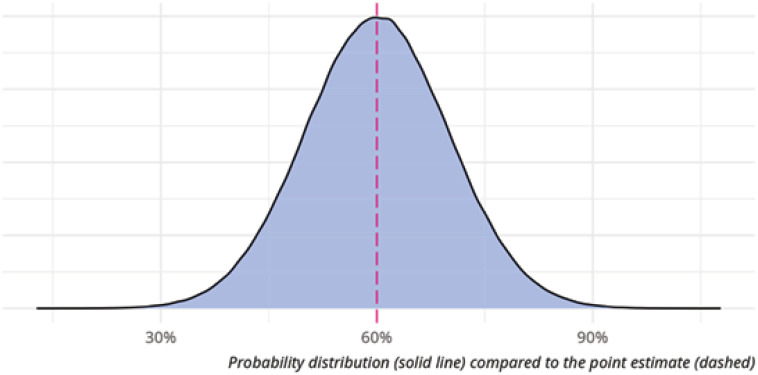
Probability distribution and point estimates.

To describe our belief in a hypothesis and to describe the hypothesis itself, we use a variety of values referred to as the parameters of a statistical model. Example parameter estimates could include the probability of a single event (a patient's illness resolving), how many events we expect to occur if we run a trial on multiple patients, and our certainty in the above two estimates being true.^(7)^ Unlike a Frequentist approach, a Bayesian statistical model does not take or produce point estimates (a single value to represent the parameter). Instead, a Bayesian model estimates probability distributions, which represent the range of possible values a parameter can take as well as how likely each value is. As an example, rather than saying the percentage chance of a single event occurring is 60%, a Bayesian model might describe a range of values from 30% to 90%, with values closer to 60% being more likely than the values at the limits of the range, as demonstrated in figure 1.

These probability distributions of parameter estimates allow us to describe what we believe to be true about a certain hypothesis. We can use probability distributions to describe our beliefs both before we gain additional information from a clinical trial and after that new information has been considered. The probability distribution describing our belief before we get new information is known as the prior distribution, and the distribution after additional information is considered is known as the posterior distribution.

#### Prior probability distributions

Our prior distribution allows us to describe our belief in a hypothesis (e.g. the size of a treatment effect) before our trial collects any new information and is based on existing knowledge, such as previous clinical or laboratory work. This is a significant criticism of Bayesian analysis, as how we interpret previous studies can be subjective.^(8)^ If done without caution, this subjective nature could push our prior distribution too far towards assuming benefit or harm, which could impact our results.

The shape of our prior distribution can be described in terms of its certainty and whether it weighed towards benefit or harm.

**Figure 2:**
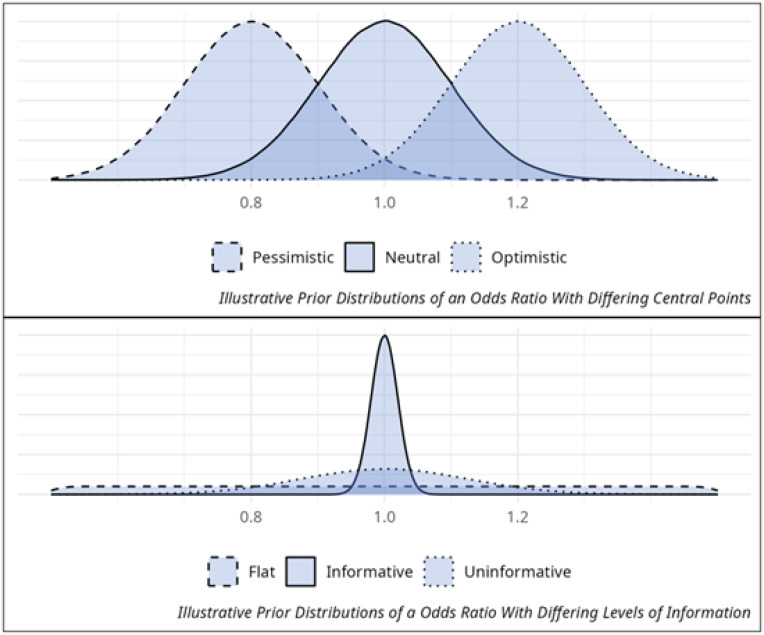
Differing shapes of priors.

If the prior distribution was centered on the most likely outcome being "no treatment effect", we could refer to this as a neutral distribution. If we increased our certainty of no treatment effect, reducing the range of possible values and decreasing the probability of benefit or harm, we would have created a more informative prior known as a skeptical distribution. In figure 2, the example informative prior is also a skeptical prior.

If we were to decrease the strength of our belief in no treatment effect, stretching the range of possible values much further into benefit and harm, we would have created an uninformative or diffuse prior. One form of an uninformative prior, which states that all outcomes are equally probable, is called a flat prior owing to the flat shape of the distribution line (Figure 2).

If pre-existing information was interpreted as suggesting a treatment effect, then most of our probability distribution would be pushed towards that, and our prior probability distribution can be referred to as an optimistic prior. Like our neutral prior, depending on the strength of belief, this distribution could be strongly or weakly informative, changing from a plot of sharp peak to a rolling hill. Finally, if we were to interpret previous information as saying there was harm from treatment, we would create a pessimistic prior distribution. Table 1 describes the key features of different types of priors.

**Table 1:** Differing types of priors

Type of prior		Description
Expected effect		
	Neutral	No difference between groups expected
	Pessimistic	Adverse treatment effect or harm expected
	Optimistic	Positive treatment effect or benefit expected
Strength of belief		
	Uninformative	Difference between groups unknown with a vast potential range in values which can approach infinity
	Informative	Previous evidence or experience suggests the results can be estimated to lie within a limited range in values and can be either neutral, positive, or negative in direction
Specific examples		
	Skeptical	A prior strongly informative of no between-group difference so that the expected effect is neutral with a limited range of values. A skeptical prior is a type of neutral prior
	Flat	Uninformative neutral prior in which any result is possible

Priors need to be predefined and justified in any analysis methods; however, more than one prior can be utilized to provide context-dependent results and additional sensitivity analysis.

#### Bayesian analysis tools

Implementing Bayesian models has become easier as computational power has increased, and more tools have been developed that support simpler specifications without resorting to implementing mathematics directly. Common examples of how modern Bayesian analysis is carried out would be with focused toolsets such as Stan and just another Gibbs sampler (JAGS) using Markov chain Monte Carlo (MCMC) approaches or an Integrated nested Laplace approximation (INLA) approach, which are usually further simplified using interfaces to these implementations built for the everyday statistical environments, such as R or Python.

The decision on what to use will generally come from familiarity and efficiency. The interface tends not to matter as you can generally find an interface to the implementation you would like to use in the statistical environment you are familiar with, for example, "Rstan" and "PyStan" for R and Python, respectively. The choice of which implementation will come down to what is likely to reduce the computation burden, speed up the process, and give the most accurate answer. Commonly JAGS is slower than Stan, which is slower than INLA. However, INLA approximates the posterior, whereas Stan and JAGS require enough samples to correctly map the posterior.^(9)^

#### Understanding Bayesian results

The Bayesian approach starts with a prior distribution and then assesses how new information generated from clinical research shapes the prior into a posterior distribution. It does this by considering the likelihood of new evidence. The Bayes theorem can concisely represent this:


$$\begin{array}{c} P(A\backslash B)=\frac{P(A\backslash B)P(A)}{P(B)}\\ \begin{array}{l} \text{Posterior probability}=\text{(likelihood x prior)}/\text{Model evidence} \end{array} \end{array}$$

Where A represents the proposed model (hypothesis), and B represents the collected data, P(A | B) is the probability that the model is correct given the collected data, P(B | A) is the likelihood that the data has been generated from the model, P(A) is your prior belief in the model estimates. Finally, P(B) is the probability that the evidence from the new data is valid.

#### Likelihood

To assess how probable it is that our hypothesis is accurate, we consider the "likelihood" of our evidence. In the context of Bayes analysis, the likelihood refers to how likely we would be to get our trial outcomes if the hypothesis described by the prior distribution was correct. Unlikely data being observed imply that our trial is a rare anomaly, and the hypothesis is still valid, or that problems with our hypothesis and our beliefs need to change.

In this likelihood description, we are talking about the probability of one thing occurring (our observed data) if another condition was met (our hypothesis being true). This type of probability is referred to as a conditional probability.

#### Posterior probability

After a study's completion, the observed evidence and the prior probability distribution are combined to form the new estimate, the posterior probability distribution. In combining the prior and likelihood, their relative sizes and strengths are considered. A trial of one thousand patients will influence how a probability distribution changes more than a trial of ten patients. Conversely, we are more certain it will be harder to shift by new evidence in a prior distribution.^(7)^

In a Bayesian analysis of a clinical trial, results are presented and interpreted using the posterior distribution. This posterior distribution may be described graphically or with summary figures; however, it is most commonly described with a mixture of point estimates and ranges.^(2,10,11)^ This posterior probability updates our previously stated prior beliefs with the newly acquired data to form our new understanding.

#### Central tendency and credible intervals

**Figure 3:**
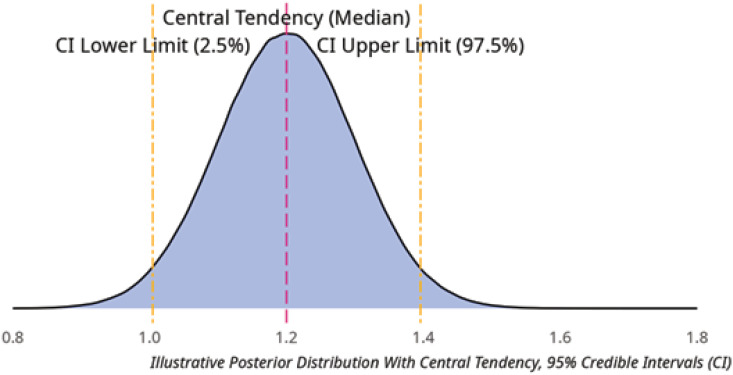
Central tendency and credible intervals.

The central tendency describes the center of the "peak" of a posterior distribution, in which the most probable estimate for the value of interest lies, as shown in figure 3. The median or mode tends to be recommended as point estimates for central tendency. The most appropriate measure depends on the nature of the posterior distribution and the form of the outcome variable.^(11)^

CI - credible interval.

If we consider the central tendency to be a way of describing our "best guess" of the true value of a model's outcome, we can describe the spread of the distribution around the central tendency using the credible interval. Common examples of central tendency measurements would be calculating the median value of the distribution or the maximum a posteriori measure, which would be the point of the posterior distribution, which is maximal density, which is the highest peak of the posterior distribution.

In the example presented in figure 3, both the median and the maximum a posteriori are concurrent; however, on a skewed distribution, they could be disparate.

Clinicians may be used to reviewing confidence intervals in Frequentist analysis and consider a 95% confidence interval to represent the range of values in which we can be 95% confident that the "true" value sits. Unfortunately, this is a misinterpretation of a 95% confidence interval and is closer to describing a 95% credible interval. A 95% credible interval describes the spread of the posterior distribution between two values, a lower and upper limit. We can say the actual value of our outcome variable sits within this upper and lower limit, with a 95% probability of being correct. In contrast, a 95% confidence interval also describes a range of values with a lower and upper limit; however, here, "95" means that if we were to repeat our experiment infinitely, 95% of the produced confidence intervals would contain the actual value of our outcome variable.^(12)^

#### Probability of effect

Similar to Frequentist trials, the outcome variable can differ between studies, with examples including estimates of the odds ratio or log odds ratio for an intervention, the absolute risk reduction (ARR), or relative risk reduction.^(2,13)^ Using measures of odds ratio or risk reduction, results would report whether an intervention or risk factor is associated with an effect on the outcome of interest.

In Frequentist trial interpretation, results can lead to debate over "statistical" *versus* "clinical significance". In this dichotomy, we see the potential scenario where an intervention has a measurable effect (is "statistically significant"), while the effect is not of enough magnitude to be relevant in a clinical context (is "clinically significant"). With Bayesian reporting, authors may report the presence of an effect regardless of magnitude and the probability of an effect size large enough to be relevant to clinical practice.

#### Probability of any effect: probability of direction

One measure of effect, regardless of size, is the probability of direction (PD). Ranging in values between zero and 100%, a PD of 95% can be interpreted as a 95% probability of a positive treatment effect; conversely, a PD of 5% would mean a 95% probability of treatment having a harmful effect. A PD of 50% would mean there is no certainty that the treatment has a positive or negative effect. Similar to a Frequentist p value, the PD does not provide any estimate of treatment effect size.^(14,15)^ The PD can provide helpful information about the probability of the presence or absence of a treatment effect. Routinely, however, clinicians wish to know the probability of an effect size large enough to be clinically meaningful.

#### Probability of "clinically significant" effect

One of the measures used to estimate the probability of a clinically significant effect size looks for the opposite of this. The Region of Practical Equivalence (ROPE) describes the probability that an intervention's effect size is below a predetermined clinical significance threshold. As clinical significance is context-dependent, the authors should provide the predefined measure of ROPE as well as the thresholds used for clinical significance and justification for these.^(2,10,11)^

ROPE is not the only measure for clinically (in)significant results. An alternative output is to report the probability that an outcome's effect size was more significant than a predetermined "minimum clinically importance difference" (MCID). If an intervention has > 50% probability of having an effect size more significant than the MCID, it might be considered as being of "potential benefit".^(13)^

A threshold of MCID set at "more probable than not" is undoubtedly a necessary bar to cross. However, it may not be sufficient to recommend specific treatments with a higher threshold of certainty required.^(13)^ There may also be value in reporting a variety of confidence thresholds in reaching MCID, as well as the probability of a treatment being associated with a larger effect size. In their recommendations for Bayesian analysis of clinical trials, Zampieri et al. recommend reporting the probability that a treatment is associated with "outstanding benefit", "severe harm", and the above measures of MCID or any effect.^(2)^

**Figure 4:**
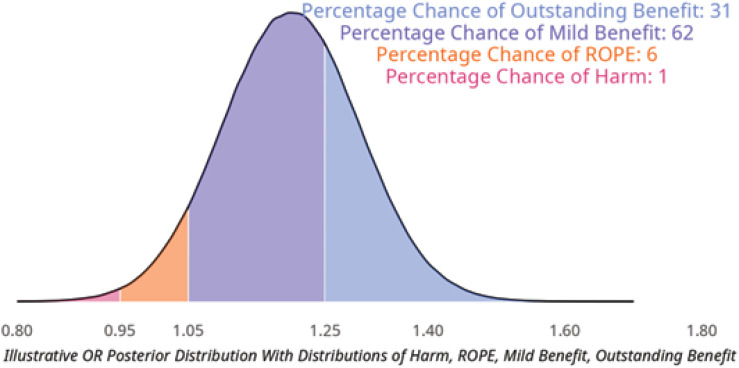
Posterior distribution split at levels of meaningful effect.

Figure 4 uses some illustrative thresholds for ROPE and clinically significant benefit when reporting the posterior estimate of the odds ratio for treatment. In this example, the percentage change of clinically significant benefit would be 93% as this is the sum of "mild" and "outstanding benefits".^(2)^

ROPE - Region of Practical Equivalence; OR - odds ratio.

While Frequentist methodologists would be expected to state their expected outcome difference *a priori*, Bayesian researchers should predefine the level of ROPE or MCID, which should be justified in the methods.

#### Null hypothesis significance testing

Within the Frequentists paradigm, the assessment and reporting of clinical trial data is usually approached using null hypothesis significance testing (NHST). This type of testing assesses how likely it would be to generate the recorded data if the null hypothesis, generally that the effect does not exist, is assumed to be true.^(16)^ With a Bayesian paradigm, given a null hypothesis, the likelihood of the data being generated can be directly compared to the likelihood of being generated by an alternative hypothesis. This can be used to quantify this difference, which is not feasible with the Frequentist methodology.

#### Prior setting and sensitivity analysis

Given the impact that the choice of prior probability distributions has on the results of a piece of research, it is unsurprising that every piece of guidance on interpreting Bayesian clinical trials recommends that the priors used be reported, explained and justified.^(2,10,11)^ Potential sources for the prior distribution have been proposed by Ferreira et al., listing: "meta-analysis, previous studies, expert opinion, or biophysical theory". They note that expert opinion could be controversial, as it introduces a more subjective element.^(12)^

#### Sensitivity analysis using multiple priors

As priors can influence results, consensus guidance is that Bayesian clinical research should report a sensitivity analysis of priors. This process demonstrates how different choices in prior selection could generate differences in estimated results.^(2,10,11)^ Recommendations tend to state that a sensitivity analysis should include some or all distributions: informative, uninformative, optimistic, pessimistic, and skeptical. If an informative prior is used, it should also be compared to other plausible informative prior distributions.^(2,10,11)^ Alternatively, an analysis that uses uninformative priors should show that another choice of uninformative priors would result in close to the same posterior distribution to demonstrate that the trial data are providing enough information for the results.^(11)^

It is worth noting that the more significant the amount of data collected by a trial, the less the prior influences the results, so the less concerned we need to be about the choice in prior. It could be argued that we should interpret a trial in which a change to the prior distribution leads to a change in our estimated results as a trial that is not yet conclusive, as the new results are insufficient to overcome changes to the prior distribution.^(10)^

Two examples of the impact of different prior settings on results from critical care studies come from the AID-ICU Trial and EOLIA study.^(5,6)^ Firstly, "Haloperidol vs. placebo for the treatment of *delirium* in ICU patients: a pre-planned, secondary Bayesian analysis of the AID-ICU trial" by Andersen-Ranberg et al.^(6)^ Their supplementary material^(6)^ both describes each decision on which priors to be set and why that prior was chosen. For the primary outcome of days alive out of the hospital, the prior for the model intercept was chosen such that the control group would have a value centred on 25 days as this was the expected result based on clinical expertise but needed to cover a range between zero and 90 days. So, a Gaussian distribution with a mean of 25 and a standard deviation of 40 was settled on as this would cover the needed range and exceed it, ranging between −53.4 and 103.4 days, but would most likely give values close to 25. This choice of a symmetric distribution, the Gaussian distribution in this case, could generate unrealistic negative values if there is not enough data to compensate for the pull of this prior. To avoid this situation, a different choice of distribution could have been made, such as a Poisson or Negative binomial, which have the inherent property that they are never negative.

Secondly, "Extracorporeal membrane oxygenation for severe acute respiratory distress syndrome and posterior probability of mortality benefit in a post hoc Bayesian analysis of a randomized clinical trial" by Goligher et al. makes use of sensitivity analyses covering prior distribution specification.^(5)^ The paper specifies a range of values defining the priors, enabling assessing how much prior choice affects the final effect estimates. The distributions range from intensely skeptical to enthusiastic, which is achieved by varying the values of the most likely estimates and overall ranges. For each distribution, a justification is given for why this value and range was chosen. For the strongly enthusiastic prior, the values were chosen to emulate a randomized control trial enrolling one hundred patients and finding a 33% relative risk reduction, and the strongly skeptical prior emulated a randomized clinical trial (RCT) enrolling 264 patients and finding a 0% relative risk reduction.

#### Model validity and predictive accuracy

Like a Frequentist analysis, a Bayesian approach should provide enough information to enable an informed judgment of how the methods have been carried out and the appropriateness of any conclusions ultimately drawn from any models produced. The main difference between the paradigms is in what should be reported. In addition to the standard assumption checking, MCMC-based Bayesian methodologies should show that the process adequately describes posterior probability distribution. This is usually achieved by reporting metrics describing the number of samples and how well they have converged and covered the probability space, such as the expected sample size (ESS) or the Rubin-Gelman R-hat value.^(11,17)^ For non-MCMC approaches such as INLA, most validation will be reliant on checking that the posterior distribution approximation aligns with the outcome distribution and that the common modelling assumptions hold.

When assessing the relevance of a model created, it is recommended that authors demonstrate some measure of model "goodness of fit". Similar to predictive models generated using non-Bayesian techniques, such a measure means showing that any predictions generated by a model are found to be correct when tested.^(11,12)^ These measures can be performed visually with plots of model predictions using "posterior predictive checks", or through measures seen in other classification problems, such as the receiver-operator curve (ROC).

#### Comparison to Frequentist methods

Accepting that shifting from one technique to another has costs, authors should be able to justify why this switch is worth undertaking, either by demonstrating the advantages of a less common approach or the disadvantages of current techniques. Table 2 summarizes some of the strengths and challenges associated with Bayes analysis compared to more standard Frequentist methods.

**Table 2:** Strengths and challenges with Bayes technique

Strengths of Bayesian analysis	
Addressing the research question	Summarising the probability distribution, including the probability of clinically meaningful effect, allows the research question to be directly answered with particular relevance to clinical practice, in a way that Frequentist use of null-hypothesis testing does not. While the p value used in Frequentist methods may be considered helpful, inappropriate interpretation is common, and there is an increased willingness to accept methodological techniques without p values
Estimating effect sizes	Bayes analysis estimates the probability of a meaningful clinical effect and the probability of dramatic benefit or associated harm
Assessing the results from different perspectives	Results from a range of priors can be interrogated and reported. On an individual patient level, different prior distributions and decision thresholds may be helpful when undertaking shared decision-making and gaining adequately informed consent
Assumptions	Justification of any assumptions made by prior distributions is routinely documented due to enhanced oversight of the new methodology
Challenges with Bayesian analysis	
Computational burden	Due to the sampling from the posterior distribution, Bayesian techniques can be costly in both time and computational burden. As the sample size of data increases, sampling becomes more burdensome. INLA, as an approach, does address some of these shortcomings
Lack of prior information	A Bayesian trial does not require information gained from previous trials, and a sensible starting point may be a neutral prior. However, if a major aspect of Bayes is using previously gained information, then a Bayesian approach may seem less relevant when no prior information is available
Lack of local expertise	The effort involved in learning a new technique may make little sense when considering other priorities. Most institutions are less familiar with Bayes, and a lack of colleagues comfortable with these techniques can increase the risk of methodological or interpretation errors
Subjective nature	Considering prior knowledge may be considered both an advantage and a disadvantage. However, for many clinicians, this aspect may be seen as an additional level of complexity and an opportunity for subjectivity to be introduced, skewing results away from the truth. Guidelines have been developed to provide reassurance by proposing a standardized choice of priors for all analyses, with only the ROPE limits left to the individual research team

INLA - Integrated nested Laplace approximation; ROPE - Region of Practical Equivalence.

A common critique of Bayesian approaches in medicine is seen when an initially Frequentist trial is re-analyzed with Bayesian techniques. Readers may perceive that the authors did not get the results they wanted, so they used a Bayesian method to get a second shot at a "positive trial."

Yarnell et al. evaluated this within the critical care literature in 2021.^(13)^ Their team investigated all multi-center trials published between 2008 and 2018 in five relevant journals, and the primary outcome was mortality in a group of critically ill patients. Identifying 82 papers, they re-performed each analysis with predetermined Bayesian techniques.^(13)^ They aimed to identify the level of disagreement between Frequentist and Bayesian techniques and any factors that increased the risk of disagreement.

The key scenarios in which the two approaches could disagree in their results were the following:

- Frequentist reported "positive", but Bayesian analysis found the probability of Minimum Clinically Important Difference (MCID) < 50%. This could be seen in which a Frequentist trial found "statistical significance" and a Bayesian trial found the PD tended to the positive, but that the most likely values of effect size sat within the ROPE.
- Frequentist reported "negative", but Bayesian analysis found a probability of MCID > 50%. If a Frequentist trial had been powered to detect a considerable ARR and failed to detect it, it may report a negative result. However, the same Frequentist trial would be "underpowered" to correctly identify a smaller ARR, one still larger than the MCID. The Bayesian reanalysis would be able to identify if there was a greater than 50% chance of this smaller but meaningful treatment effect size.^(13)^

With this second scenario of "negative Frequentist, positive Bayesian", critics of a Bayesian approach could argue that the prior distribution selected could be so biased in favor of an effect existing, it outweighs truly "negative" data. To prevent bias from optimistic priors, Yarnell's key comparison to Frequentist analyses used a skeptical prior, and the most likely effect on Absolute Risk Reduction was set at zero.^(13)^ They also performed their analysis with predetermined skeptical, uninformative, and enthusiastic priors.^(13)^ The MCID was generated for each paper by surveying a group of critical care clinicians and taking the median of their responses.

Yarnell et al. found that Bayesian reinterpretation using skeptical priors, agreed with the original Frequentist interpretation in 73 out of 82 papers (89%).^(13)^ There were different conclusions for nine studies: seven (9%) trials found to be negative under Frequentist analysis had a greater than 50% probability of meeting the threshold of MCID, and two of the four trials found to be positive under Frequentist analysis had a less than 50% probability of meeting the threshold of MCID (Table 3).

**Table 3:** Results of Yarnell's comparison of Bayesian and Frequentist randomized clinical trials^(3)^

	Bayes positive	Bayes negative	Total
Frequentist positive	2	2	4
Frequentist negative	7	71	78
Total	9	73	82

Notably, when an "enthusiastic" (optimistic) prior was used, the rate of disagreement rose substantially, with 20/78 (26%) papers being subsequently reinterpreted as "positive" by a Bayesian analysis.^(13)^

To explain the difference seen between the Bayesian reinterpretation and the original papers, Yarnell et al. found their estimates of MCID tended to be lower than what the original authors had powered their papers for.^(13)^ This implies either that original trial authors are more optimistic about the potential benefit of proposed treatments (a plausible bias, considering authors are a group of people motivated enough to perform a trial on the subject), or that original authors may be setting their estimates of effect size "pragmatically" large, to allow for a smaller sample size when recruiting. The decision to power a study to a large effect size risks a type I error occurring when the null hypothesis is rejected but a true but smaller effect exists.

## CONCLUSION

This review aimed to introduce the reader to the fundamental aspects of Bayesian inference and the concepts and terminology seen when interpreting a Bayesian research trial. In doing so, it aimed to provide the reader with a high-level understanding of a Bayesian analysis, how a trial should be reported, and what these reported methods and results actually mean. It acknowledges the difficulty of taking a different approach in clinical research and highlights some inherent trip hazards in Bayesian papers. Ultimately, it aimed to show that a Bayesian approach may allow for a more direct answer to our common questions of interest, facilitates interrogation of results in ways Frequentist approaches do not, and is a valid alternative to classical Frequentist techniques.
